# Coworker support, work–family conflict, job satisfaction, and turnover intention: female employees in post-organizational socialization

**DOI:** 10.3389/fpsyg.2025.1472977

**Published:** 2025-07-10

**Authors:** Yang Bai, Jinquan Zhou

**Affiliations:** ^1^College of Management and Economics, Tianjin University, Tianjin, China; ^2^Centre of Gaming and Tourism Studies, Macau Polytechnic University, Macau, Macao SAR, China

**Keywords:** coworker support, work–family conflict, job satisfaction, turnover intention, female employee, resorts

## Abstract

This study extends the concept of organizational socialization from coworker support to its impact on shaping work–family conflict (WFC), job satisfaction, and turnover intention. Utilizing a sample of 413 female casino dealers, it investigates the interplay between coworker support, WFC, job satisfaction, and turnover intention among female employees in the Macau casino industry, focusing on the post-organizational socialization phase. The results indicate that support from coworkers significantly decreases WFC and improves job satisfaction. Both factors mediate the relationship between coworker support and turnover intention. Furthermore, WFC positively impacts turnover intention, while job satisfaction has a negative impact. These findings highlight the importance of casino management fostering supportive coworker relationships to improve job satisfaction and employee retention, thus enhancing the effectiveness of organizational socialization. This study contributes to the organizational socialization theory and provides practical implications for managing female employees in high-pressure environments.

## 1 Introduction

Organizational socialization is how new employees learn the values, norms, and behaviors necessary to function effectively within an organization (Bauer and Erdogan, 2011; Saks and Gruman, 2014). Nevertheless, employees’ understanding of the organization and behavior will change after organizational socialization for all individuals’ careers (Torlak et al., 2024). Behavioral reinforcement theory believes that it is necessary to continuously reinforce and consolidate the collective behaviors previously required and determined by the organization (Gavetti et al., 2012). Organizations need to consolidate the achievements of individual organizational socialization (Bauer and Erdogan, 2011; Moreland and Levine, 2014). Therefore, post-organizational socialization refers to the ongoing process of adapting and integrating into an organization after the initial socialization period (Stacey, 2018). However, studying employee organizational behavior changes from the post-organizational socialization stage is still a new perspective.

Moreover, coworker support remains in the organizational socialization strategy and refers to the assistance, understanding, and encouragement employees receive from their colleagues (Ashforth et al., 2007). This support can take various forms, including emotional, informational, and instrumental. Coworker support can be a form of positive reinforcement for organizational socialization (Saks and Gruman, 2011). For example, when colleagues recognize and appreciate each other’s contributions, it reinforces positive behaviors and encourages continued collaboration and effort. Recent attention has been given to the supportive relationships that develop among coworkers, and researchers have produced evidence that coworker support can benefit workers (Sloan, 2012; Singh et al., 2019; De Clercq et al., 2020). Base Conservation of Resources Theory, coworker support is a crucial social resource that can significantly impact employees’ well-being (Sloan, 2012) and job performance (Baker and Kim, 2021). This support can be categorized into two main types: emotional and instrumental. On the one hand, emotional support involves providing empathy, care, and understanding to colleagues, which helps alleviate work-related stress (Patterer et al., 2024) and enhance job satisfaction (Koseoglu et al., 2020; Nguyen and Tuan, 2022). On the other hand, instrumental support includes practical assistance and resources that help employees perform their tasks more efficiently. Further research suggests that instrumental support from coworkers has been linked to improved job involvement and life satisfaction by facilitating a better WFC (French et al., 2018; French et al., 2018). Many studies have examined organizational socialization as an antecedent of WFC, job satisfaction, and turnover intention (Allen and Martin, 2017; Shi et al., 2023). Still, there has been no research on the impact of sustained coworker support in post-organizational socialization on the above organizational behavior outcomes.

Additionally, female newcomers often receive less social support and mentoring than their male counterparts. However, they tend to benefit more from social tactics such as mentoring and interaction, which positively influence their role outcomes (Kowtha, 2013). Compared with male employees, as female employees grow older and gain more experience, various problems will arise in matching their family life and work environment, seriously affecting their organizations’ behavioral consequences (Norling and Chopik, 2020). However, due to their female roles, female employees are given more family demands by the outside world (Greenglass et al., 1988; Allen and Martin, 2017; Zhang et al., 2020). As female employees age and gain experience, they often encounter unique challenges in balancing their family lives and work environments. These challenges can have significant implications for their behavior and overall organizational outcomes, including the incompatibility experienced by employees between their work and family roles (Allen and Martin, 2017; Shi et al., 2023), which may lead to a decrease in job satisfaction (Netemeyer et al., 1996; Bruk et al., 2002; Grandey et al., 2005; Shi et al., 2023) and an increase in turnover intention (Huyghebaert et al., 2018; Anderson et al., 2002; Mumu et al., 2021). A study on Macau casino dealers found that over half of the participants experienced depression, a quarter had anxiety, and about two-thirds reported poor sleep quality. These issues were linked to the impacts of casino employment on family life and responsibilities (Zhou et al., 2022). The Macau casino industry presents special challenges to employees’ work-family balance due to its high salaries and shift work characteristics, especially the female dealers, as the main workforce in the casino industry and tolerate enormous work pressure (Zhou and He, 2018). Their job satisfaction and retention rates directly affect the industry’s human resources stability. However, there are still gaps in the research on WFC, job satisfaction, and turnover intention of female employees under coworker support in the post-organizational socialization context.

In light of the above discussions, this article analyzes the mutual influence and mechanism between coworker support, WFC, job satisfaction, and turnover intention in post-organizational socialization. The sample of female employees from the casinos in Macau was examined to determine how WFC and job satisfaction mediate the relationship between coworker support and turnover intention. It will contribute to the theory of organizational socialization strategy and provide practical guidance for managing female employees in the Macau casino industry.

## 2 Literature review

### 2.1 Coworker support and turnover intention

Coworker support refers to the assistance and encouragement provided by colleagues in the workplace (Nazir et al., 2016; Singh et al., 2019; De Clercq et al., 2020). In the workplace, coworker support, as a work resource, is a key resource in reducing employee turnover intention (De Clercq et al., 2020; Chung et al., 2021). When unfriendly coworker relationships hinder employees from fulfilling their job responsibilities or hinder their career development, it may stimulate employees’ turnover intention (Karatepe and Olugbade, 2017). On the contrary, employees who receive coworker support have a lower intention to resign. For example, coworker support can improve the well-being of an employee by reducing stress, role conflict, and role overload (Chiaburu and Harrison, 2008; Norling and Chopik, 2020). It also improves the working environment and stimulates positive feelings and self-esteem, which gives employees more strength and energy to cope with challenges in their work (Singh et al., 2019).

Turnover intention refers to the behavior of an employee who intends to resign but has not yet taken actual action and is a key concern for organizations (Porter and Steers, 1973; Pitts et al., 2011; Li and Yao, 2022). High turnover rates can result in additional costs for recruiting, selecting, and training new members. Thus, it will disrupt the organization’s operations and incur significant costs (Tews et al., 2013; Jolly et al., 2021; McCartney et al., 2022).

Social support from supervisors and co-workers significantly influences employees’ intention to stay. Group cohesiveness and social networks also play a mediating role in enhancing retention (Sundari et al., 2024). Coworker support is essential during socialization, helping newcomers adjust to organizational norms and reducing stress and uncertainty (Singh et al., 2019). Coworker support aids new employees’ emotional and social integration (Sloan et al., 2013). The quality of relationships with co-workers and managers can significantly influence how individuals adapt to new roles or transition out of the organization (Hatmaker, 2015). Based on the Conservation of Resources Theory, De Clercq et al. (2020) found that coworker support can alleviate employees’ work stress and thus reduce their turnover intention. Based on the above discussion, the present paper assumes that coworker support positively impacts the turnover Intention of Female employees and decreases turnover intention. Hence, the following hypothesis is stated:

*H1:* Coworker support negatively impacts turnover intention.

### 2.2 Coworker support, WFC, and job satisfaction

The issue of WFC has garnered increasing attention in recent years (Namasivayam and Mount, 2004; Allen and Martin, 2017; Shi et al., 2023). WFC is a specific type of inter-role conflict that arises from the incompatibility of role pressures between the work and family domains. Conflicts in role interaction can emerge while irreconcilable contradictions arise between work and family roles (Greenhaus and Beutell, 1985; Mumu et al., 2021). Research has shown that women tend to experience a stronger conflict between these roles than men (Greenglass et al., 1988; Allen and Martin, 2017; Zhang et al., 2020).

Job satisfaction was first proposed by Hoppock (1935), who believed that psychological and physiological satisfaction with the work environment and the work itself. Recent studies have shown that employees are increasingly concerned about the work environment, personal development, and interpersonal interactions, directly or indirectly affecting their job satisfaction (Pu et al., 2024).

Furthermore, coworker support can be one of the key methods to resolve WFC (Norling and Chopik, 2020). French et al. (2018) have analyzed the relationship between social support and WFC and found that social support, especially from colleagues, can significantly reduce the degree of WFC. In Zhang et al.'s (2020) study, a sample of 764 female nurses and doctors was analyzed to reveal that colleague support, as an important component of social support, plays a crucial role in alleviating work stress caused by WFCs, reducing emotional exhaustion, and improving the psychological well-being of female workers, and can have a positive impact. Coworker support aids new employees’ emotional and social integration to enhance their well-being and job satisfaction (Yucel and Minnotte, 2017). Coworker support significantly influences job satisfaction across various occupations and industries. Coworker support directly enhances job satisfaction as employees who perceive higher support from their coworkers report greater job satisfaction (Koseoglu et al., 2020; Nguyen and Tuan, 2022). Emotionally, coworker support can enhance employees’ sense of belonging and teamwork, improving job satisfaction (Norling and Chopik, 2020; Nguyen and Tuan, 2022). In practical work, supporting colleagues can enhance teamwork, reduce work pressure, and improve job satisfaction (Macias-Velasquez et al., 2021; Gordon and Adler, 2022). Coworker support indirectly affects job satisfaction via WFC in women but not men (Matijaš et al., 2018). Based on the above reviews, the following hypothesis is stated:

*H2:* Coworker support negatively impacts WFC.

*H3:* Coworker support positively impacts job satisfaction.

### 2.3 WFC, job satisfaction, and turnover intention

Bucks studies reveal that WFC negatively correlates with job satisfaction (Netemeyer et al., 1996; Bruk et al., 2002; Grandey et al., 2005; Shi et al., 2023). The negative effects experienced by women due to WFC are more significant (Greenglass et al., 1988; Bruk et al., 2002; Ergeneli et al., 2010; Zhang et al., 2020). Greenglass et al. (1988) argued that women experience a stronger sense of conflict between work and family roles, and this WFC leads to reduced job satisfaction. Beatty (1996) found that Canadian female professionals and managers experience a decline in job satisfaction when they feel WFC. Women face greater challenges in balancing work and family responsibilities, particularly in high-stress professions such as nursing, where the negative impact of WFC on women’s job satisfaction is especially significant (Grzywacz et al., 2006; Zhang et al., 2020).

Moreover, WFC is an essential determinant of turnover intention (Huyghebaert et al., 2018). Employees feel stressed and dissatisfied when experiencing conflicts between work and family, which may lead them to consider leaving their current positions for a better work-life balance (Anderson et al., 2002; Mumu et al., 2021). Huang and Cheng (2011) found that WFC significantly impacts job stress for female employees, and this increased job stress further reinforces their intention to leave. Yildiz et al. (2021) examined the relationship between nurses’ WFC and turnover intention and found that WFC and turnover intention are both significantly correlated factors. Additionally, job satisfaction negatively impacts turnover intentions because it reduces the likelihood of employees entertaining thoughts of leaving their positions (Tett and Meyer, 1993). Bucks studies verified that higher job satisfaction will reduce employee turnover intention (Tsai and Wu, 2010; Tschopp et al., 2014). Across various contexts, such as teaching and nursing, it has been consistently demonstrated that enhancing job satisfaction can serve as a strategy to reduce employees’ turnover intention (De Cuyper et al., 2011; Zhang et al., 2022). Therefore, the following hypotheses are proposed:

*H4:* WFC negatively impacts job satisfaction.

*H5:* WFC positively influences turnover intention.

*H6:* Job satisfaction negatively impacts turnover intention.

### 2.4 Coworker support, WFC, job satisfaction, and turnover intention

The coworker relationship is one important factor that impacts turnover intention (Cotton and Tuttle, 1986). Coworker support, as an important resource in the workplace, positively impacts employee job satisfaction and organizational commitment (Singh et al., 2019; De Clercq et al., 2020; Kmieciak, 2022). According to social exchange theory (SET), employees who perceive support from their coworkers are likely to exhibit more positive organizational behaviors (Akgunduz and Eryilmaz, 2018). Similarly, coworker support also reduces employee disengagement behaviors or turnover intentions (Chung et al., 2021; Kmieciak, 2022). However, the increase in WFC may weaken the impact of coworker support on employees’ turnover intention (Zhou et al., 2022). A meta-analytic review aimed to explore the relationship between nurses’ WFCs and turnover intentions, indicating a moderate, positive, and significant relationship between WFC and turnover intention (Yildiz et al., 2021).

In addition, job satisfaction significantly reduces turnover intention across different sectors (Bright, 2021; Pu et al., 2024) and significantly negatively impacts turnover intention (Zhang et al., 2022). The coworker relationship is key to job satisfaction (Cotton and Tuttle, 1986; Yildiz et al., 2021). Job satisfaction serves as a mediator in the relationship between various forms of support and turnover intention. For example, perceived organizational support influences turnover intentions through job satisfaction (Sharif et al., 2021). Similarly, Le et al. (2023) mention job satisfaction as a mediator between family support and turnover intention. Based on the above discussions, the following hypotheses are proposed:

*H7:* WFC mediates the relationship between coworker support and turnover intention.

*H8:* Job satisfaction mediates the relationship between coworker support and turnover intention.

*H9:* WFC and job satisfaction sequentially mediate the relationship between coworker support and turnover intention.

## 3 Methodology

### 3.1 Scale

The study includes independent variables such as coworker support, work–family conflict, and job satisfaction, with turnover intention as the dependent variable. The measurement tool for perceived coworker support draws mainly from the Organizational Socialization Inventory (OSI) questionnaire developed by Taormina (1994). It results in five scales for perceived coworker support, for example, “Coworkers have helped me on the job in various ways.” The measurement tool for WFC adopts scales from the WFC questionnaires Carlson et al. (2000) and Grandey and Cropanzano (1999). It comprises four measurement indicators for WFC, for example, “My work takes time away from my family and friends.” The measurement tool for job satisfaction refers to scales by Spector et al. (2007) and Judge et al. (2020). It includes four indicators for job satisfaction, for example, “I am satisfied with my current fulfilling career.” The measurement tool for turnover intention is based on scales by Bothma and Roodt (2013) and Singh et al. (1996). It yields three measurement indicators for turnover intention, for example, “I’ve seriously considered changing organizations.”

A back-translation procedure was employed to ensure content validity (Beaton et al., 2000; Son et al., 2000). The measurement was originally developed in English and subsequently translated into Chinese by two independent professionals proficient in English and Chinese. This meticulous translation process aimed to ensure the accuracy and integrity of the instrument’s content in both languages. A 5-point Likert-type scale was adopted, ranging from strongly disagree (1) to strongly agree (5).

Partial Least Squares Structural Equation Modeling (PLS-SEM) was chosen for this study due to its suitability for explaining complex relationships and effectively addressing issues such as unacceptable solutions and factor uncertainty (Fornell and Bookstein, 1982). Additionally, SPSS 26.0 was employed as another statistical tool. The evaluation of PLS-SEM followed the recommended two-step systematic approach, beginning with the measurement model assessment and then proceeding to the structural model, as suggested by Hair et al. (2013). The research model underwent testing using Smart PLS 3.2.9.

### 3.2 Data

This study focuses on the dealers of six casino enterprises in Macau. A non-random convenience sampling method was employed for questionnaire surveys to obtain the necessary data for this research. Before the formal investigation, a pre-test analysis of the questionnaire was conducted to identify potential issues with the scale, eliminate invalid items, and enhance the reliability and validity of the scale. A pre-test was administered to 22 dealers to avoid missing data from the questionnaire. After collecting the pre-test questionnaires, IBM SPSS Statistics version 26.0 was used as an analytical tool. Cronbach’s *α* coefficient was employed to verify the reliability of the questionnaire and measure the reliability, stability, and internal consistency among items. The specific Cronbach’s α data are as follows: coworker support (0.835), WFC (0.794), job satisfaction (0.839), and turnover intention (0.811), indicating a high level of reliability for the scale used in this study.

Based on the results of the pre-test analysis, the questionnaire was appropriately revised to form the final version, which was then officially distributed. The survey team consisted of four students who served as dealers in casinos. The questionnaire survey was conducted at the casinos’ exit, utilizing closed-ended questions and random sampling in the questionnaire design. The survey period was from November 5, 2023, to December 20, 2023. 500 questionnaires were distributed, and 413 valid questionnaires were collected, resulting in an effective response rate of 82.6%.

### 3.3 Sample

Table 1 presents the demographic data for the 413 collected samples. The sample in Table 1 consists of 413 participants, with a predominance of married individuals (62.0%), followed by unmarried (35.6%), and a small group classified as “others” (2.4%). Age distribution indicates that the largest segment is between 31 and 40 years (35.4%), with 41–50 years close behind (32.0%), while those aged 21–30 years and 51 years and above represent 22.5 and 10.2%, respectively. Regarding education, 38.5% have completed high school, 24.9% hold bachelor’s degrees, 22.8% have junior high or lower education, and 13.8% possess a master’s degree or higher. Regarding employment, Melco Resorts & Entertainment Ltd. is the most represented company (30.5%), followed by Sands China Ltd. (18.9%) and others. For position tenure, a significant majority (37.3%) have been in their roles for 16 to 25 years, while 30.3% have been employed for 1 to 5 years. Overall, this sample reflects a diverse demographic with a strong representation of experienced professionals in the casino industry.

**Table 1:** Sample description (*n* = 413).

Variable	Items	Frequency	Percentage (%)
Marriage	Married	256	62.0
	Unmarried	147	35.6
	Others	10	2.4
Age	21–30 years	93	22.5
	31–40 years	146	35.4
	41–50 years	132	32
	51 years and above	42	10.2
Education	junior high school or below	94	22.8
	High school	159	38.5
	Bachelor’s degree	103	24.9
	Master’s degree or above	57	13.8
Company	Melco Resorts & Entertainment Ltd	126	30.5
	Wynn Macau Ltd.	56	13.6
	Macau Gaming Holdings Ltd.	53	12.8
	MGM China Holdings Ltd.	50	12.1
	Sands China Ltd.	78	18.9
	Galaxy Entertainment Group	50	12.1
Tenure	1 year to 5 years	125	30.3
	6 to 15 years	99	24
	16 to 25 years	154	37.3
	26 to10 years and above	35	8.5

### 3.4 Measures

Table 2 presents measurement results of reliability, including internal consistency, convergence, and discriminant validity. Each item demonstrates strong factor loadings, with WFC items ranging from 0.751 to 0.809, indicating a robust relationship between the measured aspects of WFC and the underlying construct. The Cronbach’s alpha values for WFC (0.780), JS (0.822), TI (0.796), and CS (0.826) suggest good internal consistency across these scales (Nunnally, 1978). The Composite Reliability (CR) values for each construct further affirm their reliability, with WFC (0.856), JS (0.882), TI (0.880), and CS (0.876), respectively. The Average Variance Extracted (AVE) values are satisfactory, with WFC (0.598), JS (0.693), TI (0.710), and CS (0.587), indicating that the constructs explain a significant portion of the variance in their respective items (Hair et al., 2009). Notably, the Variance Inflation Factor (VIF) values are below the threshold of 5, suggesting that multicollinearity is not a concern. Overall, the CFA results indicate that the constructs are well-defined and reliable, providing a solid basis for further analysis.

**Table 2:** Results of CFA.

Item		Loading	Cronbach α	CR	AVE	VIF
Work–family conflict (WFC)	My work takes time away from my family and friends.	0.809	0.780	0.856	0.598	1.789
	I often feel too emotionally drained to engage with my family after work.	0.754				1.610
	I often feel too tired for family activities or housework after work.	0.751				1.545
	Work approaches do not help me be a good parent or partner.	0.777				1.340
Job satisfaction (JS)	I am satisfied with my current fulfilling career	0.812	0.822	0.882	0.653	1.859
	I rarely think about quitting my job	0.791				1.720
	I am satisfied with my current job	0.861				1.977
	Most people are satisfied with the job I do	0.764				1.509
Turnover Intention (TI)	I would like a job that is better than my current one	0.848	0.796	0.880	0.710	1.685
	I’ve seriously considered changing organizations	0.841				1.750
	I seriously plan to find another job within the next year	0.839				1.644
Perceived coworker support (CS)	Coworkers have helped me on the job in various ways	0.675	0.826	0.876	0.587	1.445
	Coworkers are usually willing to offer their assistance or advice	0.789				1.680
	Most of my coworkers have accepted me as a member of this company	0.798				1.856
	My coworkers have done a great deal to help me adjust to this organization	0.721				1.912
	My relationships with other workers in this company are very good	0.838				2.159

While the average variance extracted (AVE) was higher than 0.5, we can still accept values as low as 0.4. According to Fornell and Bookstein (1982), if the AVE is less than 0.5 but the composite reliability (CR) is higher than 0.7, the convergent validity of the construct is still acceptable. The value of the square root of AVE (as shown in Table 3) was significantly higher than the correlation coefficient of the construct with other constructs, indicating that convergent validity is quite high (Hair et al., 2014). All constructs exhibited strong discriminant validity, with AVE scores > 0.5 and higher square roots of AVE than correlations with other constructs.

**Table 3:** Mean values, standard deviation, and correlation coefficient.

*N* = 413	Mean	Standard deviation	1	2	3	4	5	6	7	8	9
Marriage	2.170	0.952	1								
Age	2.300	0.930	0.695**	1							
Education	2.300	0.971	−0.274**	−0.276**	1						
Company	3.120	1.821	−0.157**	−0.149**	0.157**	1					
Tenure	2.240	0.979	0.473**	0.604**	−0.093	0.016	1				
CS	3.053	0.846	0.016	0.073	−0.004	−0.191**	0.049	**0.766**			
WFC	3.294	0.737	0.006	−0.044	0.101*	0.138**	−0.02	−0.226**	**0.773**		
JS	3.324	0.688	0.085	0.146**	−0.012	0.035	0.089	0.298**	−0.207**	**0.808**	
TI	2.645	0.836	−0.122*	−0.168**	0.09	0.084	−0.07	−0.379**	0.281**	−0.738**	**0.843**

* *p* < 0.01, ** *p* < 0.001; WFC, work–family conflict; JS, job satisfaction; TI, turnover intention; CS, perceived coworker support; The square root of AVE (bold values) is shown in parenthesis demonstrating discriminant validity.

The model was examined with the unweighted least squares discrepancy (d_ULS) value of 0.735, the geodesic discrepancy (d_G) value of 0.232, indicating covariance matrix matches the observed covariance matrix. The Normed Fit Index (NFI) value of 0.797, the Chi-square value of 568.057, and the SRMR value of 0.074 were acceptable values. Therefore, all indicators met the model’s standards, and the model fit was good.

## 4 Results

**Figure 1:**
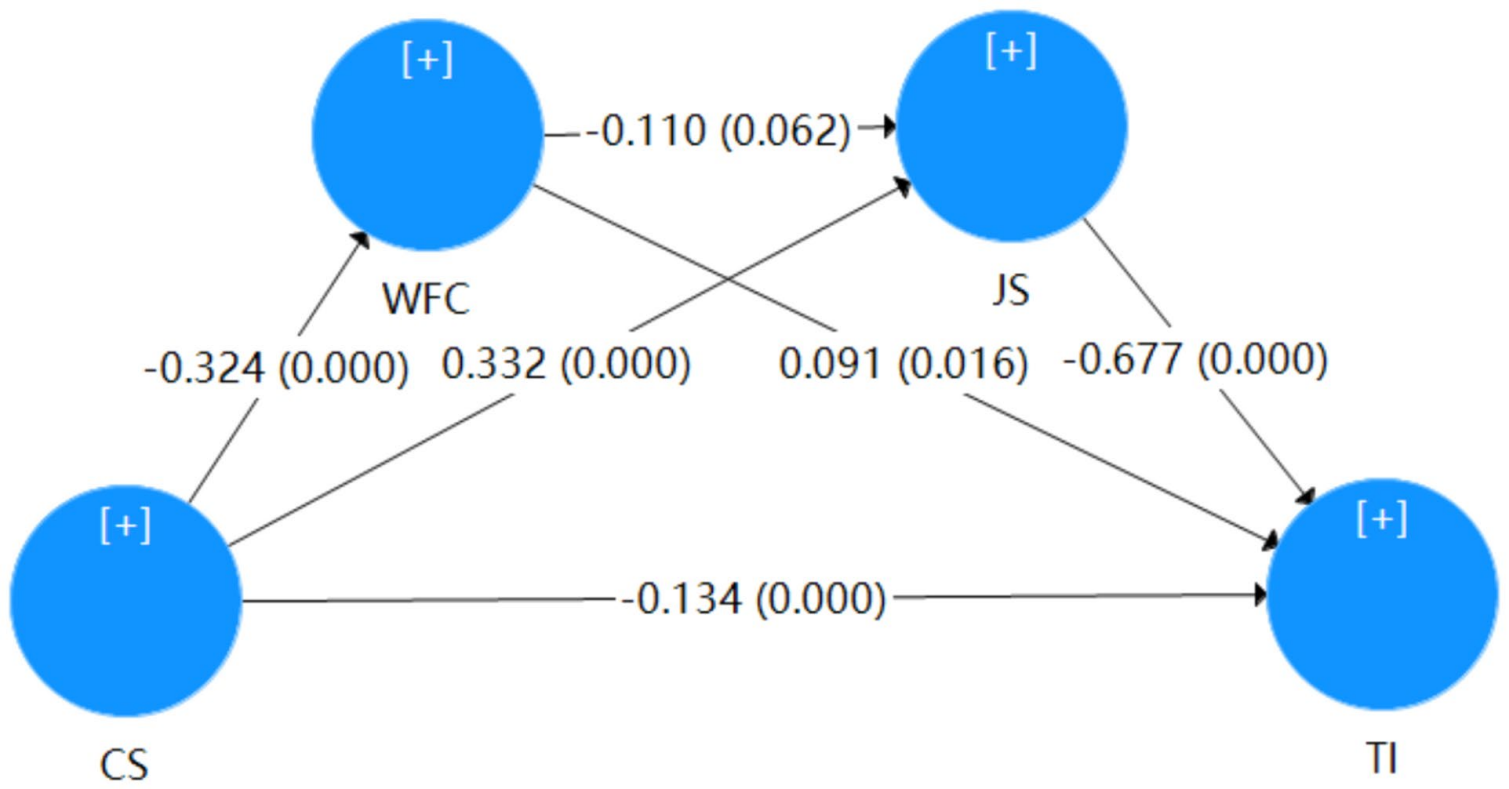
Path coefficient of the model.

Table 4 summarizes the results of the path analysis, evaluating the relationships among perceived coworker support (CS), WFC, job satisfaction (JS), and turnover intention (TI). It shows that all hypothesized paths except one are supported. Specifically, CS negatively influences TI (B = −0.134, *p* < 0.001), H1 was supported, CS significantly reduces WFC (B = −0.324, *p* < 0.001), and positively influences JS (B = 0.332, *p* < 0.001), H2 and H3 were supported. However, the path from WFC to JS is only marginally significant (B = −0.110, *p* = 0.062), H4 was unsupported, and WFC has a strong negative impact on TI (B = −0.677, *p* < 0.001), H5 was supported. Lastly, JS positively affects TI (B = 0.312, *p* < 0.001), and H6 was supported. The path coefficients for the model are shown in Figure 1.

**Table 4:** Path analysis.

Path	B	SE	*T*-value	*p* values	Decision
H1: CS → TI	−0.134	0.033	4.030	0.000	Supported
H2: CS → WFC	−0.324	0.046	7.083	0.000	Supported
H3: CS → JS	0.332	0.045	7.383	0.000	Supported
H4: WFC → JS	−0.110	0.059	1.867	0.062	Unsupported
H5: WFC → TI	−0.677	0.031	21.489	0.000	Supported
H6: JS → TI	0.312	0.057	5.479	0.000	Supported

WFC, work–family conflict; JS, job satisfaction; TI, turnover intention; CS, perceived coworker support.

Table 5 presents the results of the mediation effect analysis, exploring how perceived coworker support (CS) influences turnover intention (TI) through WFC and job satisfaction (JS). Hypothesis 7 (H7) indicates a significant mediation effect where CS negatively impacts TI through WFC (B = −0.030, *p* = 0.027); H7 was supported. Hypothesis 8 (H8) also finds strong support, with CS leading to decreased TI via enhanced job satisfaction (B = −0.225, *p* < 0.001), and H8 was supported. However, Hypothesis 9 (H9), which posits a mediation pathway of CS influencing TI through WFC and then JS, is unsupported (B = −0.024, *p* = 0.090).

**Table 5:** Mediation effect analysis.

Hypotheses	Path	B	SE	*T*-value	*p* values	Decision
H7	CS → WFC → TI	−0.030	0.013	2.208	0.027	Supported
H8	CS → JS → TI	−0.225	0.033	6.774	0.000	Supported
H9	CS → WFC → JS → TI	−0.024	0.014	1.697	0.090	Unsupported

WFC, work–family conflict; JS, job satisfaction; TI, turnover intention; CS, perceived coworker support.

## 5 Discussions and conclusions

The present study developed a model to analyze the relationship between coworker support, WFC, job satisfaction, and turnover intention. The collected sample data from full-time female employees in six casino enterprises in Macau were employed to examine all presented hypotheses. The detailed findings and conclusions are outlined as follows:

### 5.1 Discussions

Firstly, the study found that coworker support positively impacts job satisfaction, while coworker support negatively impacts turnover intention and WFC, which is consistent with previous studies (French et al., 2018; Koseoglu et al., 2020; Nguyen and Tuan, 2022). It shows that coworker support is a significant factor that positively influences job satisfaction for female dealers in the Macau casino industry. Coworker support can enhance a sense of receiving support and recognition, meet their social needs and sense of belonging, and thus improve job satisfaction. In practical work, colleague support can enhance teamwork, reduce work pressure, and improve job satisfaction (Macias-Velasquez et al., 2021; Gordon and Adler, 2022). Coworkers’ support can help female employees enhance teamwork, reduce work pressure, and improve job satisfaction (Macias-Velasquez et al., 2021; Gordon and Adler, 2022). Coworker support negatively influences turnover intention, aligning with De Clercq et al. (2020) findings. In the casino industry, female dealers face greater work pressure from factors such as high-intensity work pace, complex customer service demands, and uncertainty in career development (Zhou and He, 2018). These pressures not only affect the physical and mental health of female dealers but also significantly increase their intention to resign. However, coworker support can alleviate female dealers’ work pressure and improve their job satisfaction and organizational cohesion. When female dealers feel accepted and recognized by their colleagues, their sense of belonging to their work will increase, reducing their intention to resign. In addition, cooperation and support among colleagues can help female dealers better cope with challenges in their work, giving them more confidence when facing work pressure. Therefore, in the casino industry, enhancing coworker support is crucial for improving the retention of female employees during the post-organizational socialization process.

Secondly, the results of this study indicate that WFC positively influences turnover intention, while job satisfaction negatively impacts turnover intention. It shows that WFC is a significant factor that positively influences turnover intention (Anderson et al., 2002; Mumu et al., 2021). Female dealers often face more expectations of family responsibilities from the outside world and their families due to gender reasons. The particularity of working in the Macau casino industry exacerbates this phenomenon, making it easier for female dealers to have conflicts between their responsibilities in the workplace and their expectations at home (Zhou et al., 2022). This conflict often leads them to need to balance work and family, and when they feel that work pressure is too great and affects the care of their families, they increase their tendency to resign. Job satisfaction negatively impacts turnover intention, aligning with the findings of Zhang et al. (2022). As mentioned, the casino industry is characterized by a fast-paced work environment. Female dealers face not only workplace pressures but also expectations and demands from the outside world and their families. In this context, female dealers’ job satisfaction reduces turnover intentions and increases their willingness to remain in the industry for post-organizational socialization.

Thirdly, the results of this study demonstrate that both WFC and job satisfaction mediate the relationship between coworker support and turnover intention. Specifically, the impact of coworker support on employee turnover intention can be conveyed through WFC (Yildiz et al., 2021). Female dealers in the casino industry need to receive support and assistance from coworkers when facing high-pressure work. Coworker support reduces WFCs caused by work for female dealers, enabling them to achieve a work-family balance. Therefore, turnover intention has been reduced, which helps maintain the stability of the workforce. In addition, the results of this study reveal that the relationship between coworker support and turnover intention can be achieved through job satisfaction (Zhang et al., 2022). When female dealers in the casino industry feel positive support from their coworkers, their job satisfaction will significantly increase. Coworker support can help them cope with work pressure and enhance their sense of belonging and team cohesion. With the improvement in job satisfaction, the turnover intention of female dealers in the casino industry will significantly decrease.

In addition, the results of this study found that WFC does not significantly negatively impact job satisfaction, indicating that the factor of WFC did not influence female dealers’ perceived satisfaction with their jobs. It can be explained that antecedents of job satisfaction are various, and can be different in varied contexts. In Macao, employees in casinos earn higher incomes than those in other sectors, which attracts many residents to join the industry. Thus, WFC cannot be a key factor impacting job satisfaction. Furthermore, the findings of this study do not support the hypothesis that Work–Family Conflict (WFC) and job satisfaction sequentially mediate the relationship between coworker support and turnover intention. This can be attributed to the fact that female dealers experience heightened work stress, which leads to increased turnover intention, as noted by Zhou et al. (2022), rather than WFC itself. Therefore, while coworker support often benefits employee retention, its impact may be diminished in high-stress environments. This suggests that organizations need to enhance the nature and quality of support provided among coworkers to address the specific needs of female dealers from a family perspective.

Finally, the findings of this article reveal several important relationships regarding female dealers’ demographics and their implications for turnover intention and job satisfaction. Specifically, age and marital status correlate negatively with turnover intention, suggesting that elderly and married female dealers are less likely to consider leaving their jobs. Moreover, age also shows a positive correlation with job satisfaction, which implies that, like organizational socialization, they may find more fulfillment and contentment in their roles. On the other hand, education and company affiliation are positively correlated with WFC. This indicates that female dealers with higher education levels and those employed by certain companies may face greater challenges balancing their professional and personal lives. Additionally, company affiliation has a negative correlation with perceived coworker support. This suggests that female dealers within certain companies may feel less supported by their colleagues, which could further exacerbate feelings of isolation or stress, particularly in high-pressure environments. Understanding these dynamics is crucial for post-organizational socialization aiming to improve employee retention and satisfaction by fostering a supportive workplace culture.

### 5.2 Theoretical implications

The theoretical contributions of this study to the concept of post-organizational socialization lie in its exploration of the role of coworker support, work–family conflict, job satisfaction, and turnover intention within the context of the Macau casino industry, particularly for female employees. By focusing on post-organizational socialization, the study extends the existing literature on socialization processes by highlighting how these post-socialization factors influence employees’ adaptation and integration into the organization after initial onboarding. Specifically, the study underscores the mediating role of job satisfaction and work–family conflict in the relationship between coworker support and turnover intention, which has not been extensively explored in previous research. Previous studies have emphasized the importance of coworker support in reducing work-related stress and increasing job satisfaction (e.g., French et al., 2018; Koseoglu et al., 2020), but this study contributes to the theoretical literature by highlighting that coworker support influences turnover intention through both WFC and job satisfaction. The findings suggest that coworker support, by enhancing job satisfaction and reducing work–family conflict, significantly contributes to dropping turnover intention, thus promoting the stability of the workforce during the post-socialization phase. This contributes to a more nuanced understanding of post-organizational socialization by emphasizing the importance of interpersonal relationships and work-life balance in employee retention and long-term organizational commitment.

Moreover, the findings challenge some established assumptions regarding the direct impact of WFC on job satisfaction. While the literature often suggests that WFC has a detrimental effect on job satisfaction (e.g., Allen et al., 2000), this study did not find a significant negative impact of WFC on job satisfaction. This divergence could be attributed to the unique characteristics of the casino industry, where higher income levels and other contextual factors might buffer the typical negative consequences of WFC. This implies that future research should consider contextual variables, such as industry-specific factors or demographic influences, when assessing the broader applicability of WFC theories.

Finally, this study clarifies the mediating mechanism by which coworker support reduces turnover intention by alleviating WFC and increasing job satisfaction, providing a new perspective for understanding the complex role of coworker support in organizational behavior and laying the foundation for future research on the relationship between coworker support and other organizational variables.

### 5.3 Practical implications

This study offers valuable insights for organizational leaders, human resource managers, and policymakers in the casino industry, particularly those concerned with employee retention and job satisfaction. The discovery that support from coworkers significantly reduces turnover intention and alleviates work–family conflict has important implications for workplace interventions. For casino operators, fostering a supportive work environment where employees feel recognized, valued, and assisted by their colleagues could be a key strategy for retaining female employees, particularly in high-stress roles of a casino dealer. Interventions such as team-building activities, mentoring programs, and promoting a collaborative work culture may improve coworker support and reduce work–family conflict.

Secondly, the study highlights the importance of addressing WFC, particularly for female dealers who may face greater familial expectations. Casino operators should consider implementing policies that support work-life balance, such as flexible scheduling, childcare assistance, or more generous family leave policies, to help mitigate the negative impacts of WFC. This will enhance job satisfaction and decrease turnover intention among female dealers who may be balancing demanding work schedules with family obligations.

In addition, the study emphasizes the importance of increasing job satisfaction in reducing turnover intention. Casino operators should improve the working environment, optimize compensation and benefits, and provide career development opportunities to enhance dealers’ job satisfaction and identification, making them more willing to stay in the company for the long term.

Lastly, the study’s finding that certain demographic factors, such as age, marital status, and education level, influence turnover intention and WFC provides practical guidance for tailoring organizational policies. For example, older and married female dealers may benefit from specific retention strategies focused on job satisfaction and work-life balance. In contrast, younger or less experienced dealers might need extra support and training to handle the challenges of the casino environment. Understanding these demographic nuances can help managers design effective interventions and improve employee retention.

### 5.4 Conclusion

In conclusion, this study underscores the critical role of coworker support in enhancing job satisfaction and reducing turnover intention among female dealers in the Macau casino industry. The findings reveal that coworker support not only mitigates work–family conflict (WFC) but also fosters a sense of belonging, which is essential for employee retention. While WFC was found to influence turnover intention, it did not significantly impact job satisfaction, highlighting the unique context of the casino environment where higher income levels may buffer typical negative effects.

Moreover, the mediating roles of job satisfaction and WFC in the relationship between coworker support and turnover intention provide new insights into post-organizational socialization. This study emphasizes the need for casino operators to cultivate a supportive workplace culture and implement policies that promote work-life balance, ultimately enhancing employee satisfaction and stability within the workforce. By addressing the specific needs and challenges faced by female dealers, organizations can significantly reduce turnover intention and improve overall employee well-being.

## 6 Limitations

Several limitations of this study should be acknowledged. First, the sample is limited to full-time female employees in six casino enterprises in Macau, which may restrict the generalizability of the findings to other industries or regions with different cultural, economic, or organizational contexts. Expanding the sample to include male employees or those from other sectors could enhance the external validity of the results. Second, the cross-sectional design limits the ability to draw causal inferences between the variables. A longitudinal or experimental design would provide stronger evidence of causality by examining how coworker support, work–family conflict, job satisfaction, and turnover intention evolve. Third, the study relies on self-reported data, which may be subject to biases like social desirability or recall bias, and future research could benefit from incorporating objective measures such as turnover records or supervisor assessments. Fourth, while important demographic variables such as age, marital status, and education level are considered, other factors such as personality traits, leadership styles, and organizational culture may also influence the relationships studied, warranting further exploration of these variables. Finally, the study does not explore industry-specific factors that may uniquely impact the work-family dynamics and job satisfaction of female employees in the casino industry, such as irregular working hours, job security, or career advancement opportunities, which could mitigate the effects of work–family conflict and turnover intentions.

## Data Availability

The original contributions presented in the study are included in the article/supplementary material, further inquiries can be directed to the corresponding author.
